# Differences in clinical outcomes between HER2-negative and HER2-positive luminal B breast cancer

**DOI:** 10.1097/MD.0000000000034772

**Published:** 2023-08-25

**Authors:** Byeongju Kang, Jeeyeon Lee, Jin Hyang Jung, Wan Wook Kim, Heejung Keum, Ho Yong Park

**Affiliations:** a Department of Surgery, School of Medicine, Kyungpook National University, Kyungpook National University Chilgok Hospital, Daegu, Republic of Korea.

**Keywords:** breast cancer, luminal B, survival

## Abstract

The clinical features and prognosis of breast cancer can vary widely, depending on the molecular subtype. Luminal B breast cancers are usually either estrogen receptor-positive and/or progesterone receptor-positive with high proliferation of Ki67 index, or HER2 positive (HER2+). The authors compared the clinicopathologic factors and survival rates of different subtypes of luminal B breast cancer according to HER2 status. Between 2009 and 2013, 1131 cases of breast cancer were reviewed and characterized as 1 of 4 different molecular subtypes based on their immunohistochemical results: luminal A, luminal B, HER2+, or triple-negative breast cancer. From these, luminal B breast cancers were extracted and the clinical features and prognosis of the HER2- and the HER2 + subtypes were compared. Survival differed significantly based on the molecular subtype regardless of whether or not the patient received treatment with neoadjuvant chemotherapy. While patients with HER2- luminal B breast cancer who received neoadjuvant chemotherapy had better prognoses, patients with HER2 + luminal B breast cancer who did not receive neoadjuvant chemotherapy had better prognoses. Luminal B breast cancers showed different clinical outcomes and survival rates according to HER2 gene overexpression type. Physicians should consider these results when they establish a treatment strategy.

## 1. Introduction

Breast cancer is a heterogeneous disease that shows different behaviors according to clinical and pathologic factors as well as molecular subtypes.^[1–3]^ When classified by gene expression profiles, breast cancers can be divided into the following types: luminal A and luminal B breast cancers, HER2-enriched breast cancers, and basal-like breast cancers.^[4,5]^ Breast cancer is categorized because the appropriate treatment approach and potential outcomes vary depending on the specific type of cancer.

The molecular subtype of breast cancer can only be precisely identified through genetic profiling. However, they can be roughly classified using immunohistochemical (IHC) biomarkers, because there is a high correlation between the molecular subtypes and IHC results.^[6–11]^ Luminal breast cancer is usually characterized by signal expression of receptors for estrogen receptor-positive (ER + ) or progesterone receptor-positive (PR + ), with either low proliferation of Ki67 index (A-type) or high proliferation of Ki67 index (B-type). Additionally, ER + and/or PR+, HER2 + breast cancers are classified as another type of luminal B breast cancer.^[5,12]^ Therefore, luminal B breast cancers can be either ER + and/or PR + , HER2−, high Ki67 index, or ER + and/or PR + , HER2 + , with either high or low Ki67. Because they have different molecular signals, the treatment and prognosis for these 2 types are different, even if both are referred to as luminal B breast cancer.

The authors analyzed the clinical outcomes and prognosis of breast cancer based on the molecular subtypes and compared the oncologic outcomes of the different subcategories of luminal B breast cancer.

## 2. Methods

A total of 1131 patients with breast cancer who underwent surgery and additional treatments at the Kyungpook National University Hospital (Daegu, Republic of Korea) between 2009 and 2013 were included in this study. The mean follow-up period was more than 8 years, and all breast cancer-specific events were recorded. Clinical records and pathologic results were all reviewed and analyzed, and the cancers were classified into 4 different molecular subtypes based on their IHC biomarkers: luminal A, luminal B, HER2 + , and tipple-negative breast cancer (TNBC). The Ki67 proliferation index was considered high when > 15% of tumor cells showed nuclear immunoreactivity. The criteria in the ASCO/CAP 2016 guidelines were followed for the histopathological examination of 4 biomarkers.

All luminal B breast cancers were categorized into 2 subgroups: ER + and/or PR TNBC + , HER2−, high Ki67 proliferation index (referred to as *HER2- luminal B breast cancer*) and ER + and/or PR + , HER2 + (referred to *HER2* + *luminal B breast cancer*). The 2 subgroups were compared according to clinicopathologic characteristics and oncologic outcomes. The analysis also included identifying whether or not patients with luminal B breast cancer had received neoadjuvant chemotherapy (NAC).

Clinicopathologic variables included the patient’s age at breast cancer diagnosis, body mass index, clinical and pathologic tumor size, clinical lymph node status, neoadjuvant or adjuvant treatment status, and oncologic outcomes. If patients received NAC before surgery, the pathologic complete response (pCR) for breast lesions and axillary lymph nodes was also evaluated using mammography, ultrasonography, breast MR, and chest/abdominal CT.

All procedures in this study that involved human participants were performed in accordance with the ethical review of the Institutional Review Board of the Kyungpook National University Chilgok Hospital (KNUCH 2015-05-205). The experimental protocol was also approved by the Institutional Review Board of the Kyungpook National University Chilgok Hospital and all experiments were performed in accordance with relevant guidelines and regulations.

## 3. Statistical analysis

All statistical analyses were performed using SPSS ver. 22.0 (SPSS, Chicago, IL). Categorical variables were analyzed using the chi-squared test in univariate analysis, and oncologic outcomes were assessed using Kaplan–Meier analysis to identify factors affecting locoregional recurrence, distant metastasis, or death. A *P* value < .05 was considered to be statistically significant.

## 4. Results

**Figure 1.**
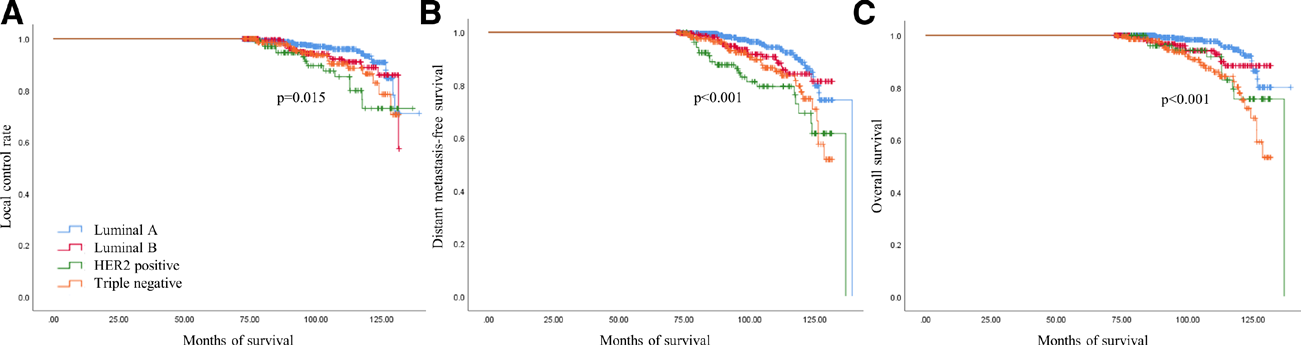
Survival analysis according to the molecular subtypes with local control rate (A), event-free survival (B) and overall survival (C).

For the 1131 patients, the breast cancers were classified into the following molecular subtypes: luminal A (n = 551), luminal B (n = 251), HER2 + breast cancer (n = 111), and TNBC (n = 218). The clinical variables (patient’s age at diagnosis, body mass index, clinical tumor size, pathologic tumor size, and clinical lymph node status) did not show significant differences between the 4 groups. Different treatment strategies (NAC, adjuvant chemotherapy, and radiotherapy) had different outcomes, however, based on the tumor characteristics. HER2 + breast cancers and TNBC cancers showed worse oncologic outcomes than the luminal type breast cancers (locoregional recurrence (*P* = .015); distant metastasis (*P* < .001); death (*P* < .001) (Fig. 1).

**Figure 2.**
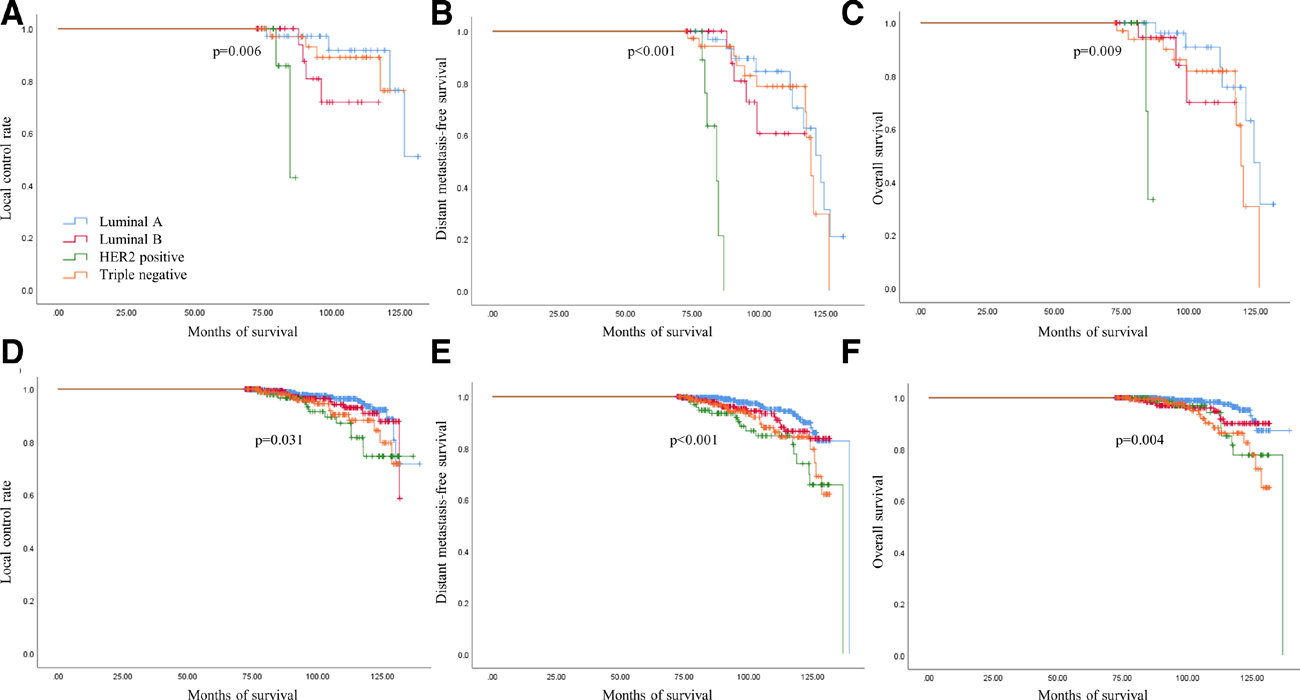
Survival analysis of patients with breast cancer according to the molecular subtypes with local control rate, event-free survival and overall survival. (A–C) Survivals in patients with breast cancer who received the neoadjuvant chemotherapy. (D–F) Survivals in patients with breast cancer who did not receive the neoadjuvant chemotherapy.

In patients who received NAC, the rate of pCR in the breast and axillary lymph nodes was higher for HER2 + breast cancer and TNBC than for the luminal breast cancer types (*P* = .047, 0.020) (Table 1). The incidence of locoregional recurrence, distant metastasis, and death in patients with breast cancer all differed significantly according to molecular subtype regardless of NAC treatment status (Fig. 2).

**Table 1:** Clinicopathologic characteristics of patients with breast cancer according to the molecular profile.

	Subtypes				
Total (n = 1131)	Luminal A (n = 551)	Luminal B (n = 251)	Her2 positive (n = 111)	Triple-negative (n = 218)	*P* value
Age at diagnosis (mean ± SD, yr)	49.92 ± 10.90	49.74 ± 10.60	50.47 ± 9.79	49.51 ± 10.52	.936
Body mass index (mean ± SD, kg/m^2^)	23.63 ± 3.44	23.77 ± 3.04	23.58 ± 3.29	23.88 ± 3.36	.777
Clinical tumor size (mean ± SD, cm)	2.21 ± 1.54	2.76 ± 1.93	3.62 ± 2.38	2.71 ± 1.74	.100
Pathologic tumor size (mean ± SD, cm)	1.80 ± 1.02	2.0 ± 1.48	1.72 ± 1.16	1.80 ± 1.22	.059
Clinical lymph node status (n, %)					.064
Negative	365 (66.25)	155 (61.76)	75 (67.57)	160 (73.40 (9)	
Positive	186 (33.75)	96 (38.24)	36 (32.44)	58 (26.61)	
Neoadjuvant chemotherapy (n, %)	38 (6.90)	26 (10.36)	11 (9.91)	37 (16.98)	<.001
pCR* in breast (n, %)	4/38 (10.53)	3/26 (11.54)	4/11 (36.37)	9/37 (24.33)	.047
pCR in axillary lymph node (n, %)	3/38 (7.90)	0	2/99 (18.19)	6/37 (16.22)	.020
Adjuvant chemotherapy (n, %)	274 (49.73)	175 (69.73)	88 (79.28)	153 (70.19)	<.001
Adjuvant radiotherapy (n, %)	350 (63.52)	149 (59.37)	54 (48.65)	137 (62.85)	.208
Follow-up period (mean ± SD, months)	97.84 ± 16.90	98.80 ± 17.18	99.51 ± 15.09	99.40 ± 16.50	.592
Locoregional recurrence (n, %)	22 (4.00)	16 (6.38)	14 (12.62)	18 (8.26)	.015
Distant metastasis (n, %)	32 (5.81)	21 (8.37)	23 (20.72)	30 (13.77)	<.001
Death (n, %)	21 (3.82)	18 (7.18)	17 (15.32)	27 (12.39)	<.001

* pCR, Pathologic complete response.

There were 26 luminal B breast cancer patients who received NAC and 225 patients who did not receive NAC. The clinicopathologic variables of the patients with luminal B breast cancer are shown in Table 2. When the luminal B breast cancers were divided into HER2− and HER2 + groups, the pathologic tumor size after NAC was significantly larger in the HER2− luminal B breast cancer group (*P* = .001). After NAC, pCR was only achieved in the HER2−, luminal B breast cancer group. Therefore, HER2 + luminal B cancer patients who had received NAC were more likely to undergo additional chemotherapy after surgery (*P* = .014). The incidence of both locoregional recurrence and distant metastasis was higher in the HER2 + luminal B breast cancer group who had received NAC (*P* = .001, 0.001).

**Table 2:** Clinicopathologic characteristics of patients with Luminal B-type breast cancer.

		Luminal B-type breast cancer with neoadjuvant chemotherapy (n = 26)	Luminal B-type breast cancer without neoadjuvant chemotherapy (n = 225)
Age at diagnosis (mean ± SD, yr)		50.00 ± 7.54	49.71 ± 10.37
Body mass index (mean ± SD, kg/m^2^)		24.92 ± 3.30	23.64 ± 2.98
Clinical tumor size (mean ± SD, cm)		5.01 ± 2.01	2.44 ± 1.69
Pathologic tumor size (mean ± SD, cm)		2.70 ± 2.65	1.95 ± 1.29
Clinical lymph node status (n, %)	Negative	4 (15.39)	
	Positive	22 (84.61)	
Estrogen receptor status (n, %)	Positive	21 (80.77)	187 (83.12)
	Negative	5 (19.23)	38 (16.89)
Progesterone receptor status (n, %)	Positive	17 (65.39)	128 (56.89)
	Negative	9 (34.62)	97 (43.12)
HER2/*neu* gene status (n, %)	Positive	7 (26.93)	108 (48.00)
	Negative	19 (73.08)	117 (52.00)
Ki67 index (n, %)	< 15 %	8 (30.77)	78 (34.67)
	≥ 15%	18 (69.23)	147 (65.34)
Adjuvant chemotherapy (n, %)		15 (57.70)	160 (71.12)
Adjuvant radiotherapy (n, %)		22 (84.62)	127 (56.45)
Adjuvant hormone treatment (n, %)		25 (96.16)	205 (91.12)
Follow-up period (mean ± SD, months)		90.39 ± 12.62	99.77 ± 17.39
Locoregional recurrence (n, %)		4 (15.39)	12 (5.34)
Distant metastasis (n, %)		5 (19.23)	16 (7.12)
Death (n, %)		3 (11.54)	10 (4.45)

**Figure 3.**
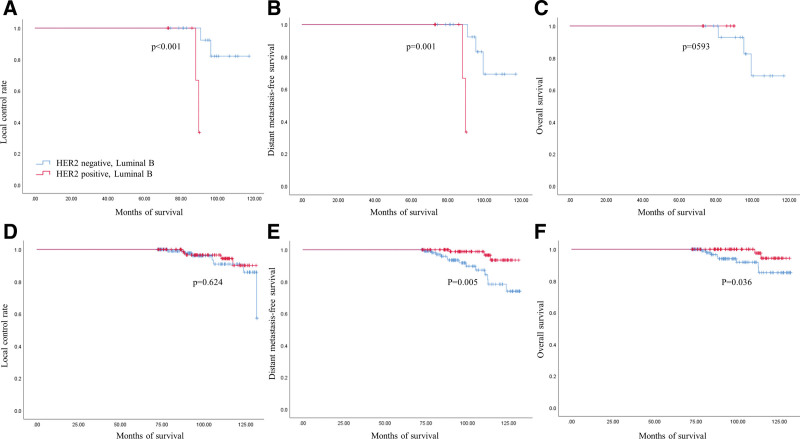
Comparison of survivals in patients with Luminal B breast cancer between HER2 negative and HER2 positive. (A–C) Survivals in patients with breast cancer who received the neoadjuvant chemotherapy. (D–F) Survivals in patients with breast cancer who did not receive the neoadjuvant chemotherapy.

In patients with luminal B breast cancer who did not receive NAC, adjuvant chemotherapy was more frequently used in the HER2 + luminal B breast cancer group, and the incidence of distant metastasis and death were higher in the HER2− luminal B breast cancer group (*P* = .005, 0.036) (Table 3) (Fig. 3).

**Table 3:** Comparison of clinicopathologic characteristics between HER2 negative and HER2 positive Luminal B-type breast cancer.

	Luminal B-type breast cancer with neoadjuvant chemotherapy (n = 26)		*P* value	Luminal B-type breast cancer without neoadjuvant chemotherapy (n = 225)		*P* value
	HER2 negative (n = 19)	HER2 positive (n = 7)		HER2 negative (n = 117)	HER2 positive (n = 108)	
Age at diagnosis (mean ± SD, yr)	50.27 ± 8.00	49.43 ± 7.00	.884	52.09 ± 10.36	46.89 ± 9.72	.101
Body mass index (mean ± SD, kg/m^2^)	24.97 ± 3.65	24.80 ± 2.51	.134	24.28 ± 2.80	22.94 ± 3.04	.134
Clinical tumor size (mean ± SD, cm)	4.56 ± 1.84	6.24 ± 2.06	.331	2.48 ± 1.89	2.38 ± 1.43	.704
Pathologic tumor size (mean ± SD, cm)	3.02 ± 2.60	2.13 ± 2.87	.001	1.96 ± 1.28	1.94 ± 1.31	.906
Clinical lymph node status			.870			.506
Negative	2 (10.53)	2 (28.58)		73 (62.40)	72 (66.67)	
Positive	17 (89.47)	5 (71.43)		44 (37.61)	36 (33.33)	
Breast pCR after neoadjuvant chemotherapy (n, %)	4 (21.06)	0	.002	-	-	
Lymph node pCR after neoadjuvant chemotherapy (n, %)	2 (10.53)	0	.060	-	-	
Adjuvant chemotherapy (n, %)	8 (42.11)	7 (100.00)	.014	74 (63.25)	86 (79.63)	.047
Adjuvant radiotherapy (n, %)	17 (89.48)	5 (71.43)	.801	72 (61.54)	55 (50.93)	.092
Adjuvant hormone treatment (n, %)	19 (100.00)	6 (85.72)	.314	109 (93.17)	96 (88.89)	.139
Mean follow-up period (mean ± SD, months)	93.60 ± 12.54	81.68 ± 8.37	.759	97.39 ± 18.20	102.35 ± 16.17	.446
Locoregional recurrence (n, %)	2 (10.53)	2 (28.58)	.001	7 (5.99)	5 (4.63)	.684
Distant metastasis (n, %)	3 (15.79)	2 (28.58)	.001	13 (11.12)	3 (2.78)	.005
Death (n, %)	3 (15.79)	0	.593	8 (6.84)	4 (3.71)	.036

pCR = pathologic complete responses.

## 5. Discussion

Breast cancers have different prognoses and clinical implications based on their molecular subtypes.^[13–15]^ Generally, the luminal type of breast cancer has been reported to have a better prognosis than either HER2 + breast cancer or TNBC. However, luminal types A and B have different clinical outcomes because they have different degrees of Ki67 proliferation, which is an important prognostic factor in breast cancer. Similarly, there would be differences between HER2− and HER2 + luminal B breast cancers based on the expression of the HER2/neu gene.

HER2 + luminal B breast cancers can have the characteristics of both hormone-positive breast cancer and HER2 + breast cancer. A category, triple-positive breast cancer, has been reported to be less responsive to both endocrine treatment and target therapy.^[16–18]^

In many clinical studies, luminal B breast cancer is considered as a single type when analyzing clinical relevance and outcomes.^[4,5,19–21]^ However, because the treatment strategies are different for HER2− and HER2 + luminal B breast cancers, they should be analyzed as 2 distinct types. Parise CA and Caggiano V reported that the ER−, PR + , HER2 + luminal B breast cancer had the worst prognosis of the following 3 categories of HER2 + luminal B breast cancer: ER + , PR + , HER2 + (triple-positive breast cancer), ER + , PR−, HER2 + breast cancer, and ER−, PR + , HER2 + breast cancer.^[22]^ In this study, as previously known, the clinical outcomes and prognosis were significantly different according to the molecular subtypes, and luminal type breast cancer generally had a better prognosis than HER2 + breast cancer and TNBC. Especially, in patients with breast cancer who received NAC, the pCR rate was significantly higher in HER2 + breast cancer and TNBC which have higher responsiveness to chemotherapy. And the survival outcomes between HER2− and HER2 + luminal B types of breast cancer were significantly different.

The analysis was conducted by dividing the patients into groups based on whether they had received NAC, considering the initial stage of each group. NAC was used in 6.9 % of luminal A-type (n = 38), 10.4 % of luminal B-type (n = 26), 9.9 % of HER2 + breast cancer type (n = 11), and 17.0 % of TNBC type (n = 37). Of the 26 patients with luminal B breast cancer who received NAC, pCR was only identified in the HER2− luminal B breast cancer patients and these patients had a better prognosis than the HER2 + luminal B breast cancer patients. However, these patients were diagnosed with breast cancer 7 to 10 years ago and the treatment strategy for HER2 + breast cancer has evolved since then. Therefore, further analysis is needed for patients with HER2 + breast cancer who received newer treatment modalities for HER2 + breast cancer.

It has been reported that NAC can increase the pCR rate in HER2-positive and TNBC, leading to significantly improved survival rates for patients.^[23]^ The role of NAC has been further expanded within breast cancer subtypes, and even the application of immunotherapy for breast cancer has led to more improved survival results.^[24]^ However, the role of NAC in the luminal B subtype is still not fully understood. Although there were only 26 patients with luminal B-type breast cancer who received NAC in this study, it is clear that the prognosis of HER2 + and HER2− luminal B-type breast cancer is significantly different under standard of care. Physicians need to understand that the prognosis can vary depending on the molecular expression, even if the cancers are classified into the same subtype, to establish individualized treatment strategies.

## 6. Conclusion

The prognosis for patients with luminal B breast cancer differs according to HER2 status. While the HER2- luminal B breast cancer had a better prognosis in patients who received neoadjuvant chemotherapy, the HER2 + luminal B breast cancer had a better prognosis in patients who did not receive neoadjuvant chemotherapy. These results provide clinical implications for establishing the most appropriate treatment strategy for luminal B breast cancer.

## Author contributions

**Conceptualization:** Jeeyeon Lee, Byeongju Kang, Ho Yong Park.

**Data curation:** Jeeyeon Lee, Byeongju Kang.

**Formal analysis:** Jeeyeon Lee.

**Investigation:** Jeeyeon Lee, Byeongju Kang, Heejung Keum.

**Methodology:** Jeeyeon Lee, Byeongju Kang.

**Resources:** Jeeyeon Lee, Heejung Keum.

**Software:** Jeeyeon Lee.

**Supervision:** Jin Hyang Jung, Wan Wook Kim, Ho Yong Park.

**Validation:** Byeongju Kang, Jin Hyang Jung, Wan Wook Kim, Ho Yong Park.

**Visualization:** Jeeyeon Lee, Byeongju Kang.

**Writing – original draft:** Jeeyeon Lee, Byeongju Kang.

**Writing – review & editing:** Jin Hyang Jung, Wan Wook Kim, Heejung Keum, Ho Yong Park.
